# Comparison between lumbar plexus block and fascia iliaca block in hip surgery: A systematic review and meta-analysis

**DOI:** 10.1097/MD.0000000000043744

**Published:** 2025-09-05

**Authors:** Jing Wu, Hongxia Mou, Xiaowei Luo

**Affiliations:** a Department of Anesthesiology, The First Affiliated Hospital of Traditional Chinese Medicine of Chengdu Medical College, XinDu Hospital of Traditional Chinese Medicine, Chengdu, China.

**Keywords:** comparison, fascia iliaca block, hip surgery, lumbar plexus block, meta-analysis

## Abstract

**Background:**

With ultrasound-guided nerve block technology being increasingly used in hip surgery, the choice between fascia iliaca block (FIB) and lumbar plexus block (LPB) is still inconclusive. This study aims to evaluate the advantages and disadvantages of FIB and LPB in hip surgery.

**Methods:**

PubMed, Web of Science, Cochrane Library, Embase, and CNKI were searched from inception to October 4, 2022. Two authors independently screened literature, extracted data, assessed study quality, and conducted meta-analysis using Review Manager 5.4.1. The heterogeneity was assessed by *I*^2^, and the fixed-effects model was applied when *P* > .05 and *I*^2^ < 50%; otherwise, the random-effects model was applied. For dichotomous variables, relative risk (RR) with 95% confidence interval (CI) was calculated. For the measured data, the standardized mean difference (SMD) with 95% CI were calculated, and statistical significance was set at *P* ≤ .05. Sensitivity analysis was performed by comparing results between fixed- and random-effects models.

**Results:**

In this comparative study of 639 patients (FIB group, *n* = 323; LPB group, *n* = 316) undergoing general anesthesia, 21 indices were analyzed via meta-analysis, with 12 showing heterogeneity and 7 lacking stability. FIB demonstrated superiority in ultrasound imaging time [SMD = −1.53, 95% CI (−1.93 to −1.13), *P* < .001], puncture time [SMD = −3.02, 95% CI (−4.12 to −1.91), *P* < .001], and length of stay [SMD = −0.43, 95% CI (−0.78 to −0.08), *P* = .02]. LPB outperformed in time to take effect [SMD = 1.76, 95% CI (0.13–3.39), *P* = .03], end-of-operation heart rate [SMD = 0.55, 95% CI (0.18–0.91), *P* = .03] and blood pressure [SMD = 0.88, 95% CI (0.51–1.26), *P* < .001], intraoperative sufentanil dose [SMD = 2.22, 95% CI (0.84–3.59), *P* = .002], 24-hour postoperative sufentanil dose [SMD = 1.80, 95% CI (0.17–3.42), *P* = .03], and postoperative 1-hour visual analogue scale (VAS) score [SMD = 0.48, 95% CI (0.16–0.80), *P* = .003]. No significant differences were observed in hemodynamics during laryngeal mask implantation or skin incision, remifentanil dose, patient-controlled analgesia (PCA) usage time, postoperative VAS scores at 6, 8, 12, 24, 48 hours, or adverse event incidence.

**Conclusion:**

LPB significantly reduced intraoperative and postoperative opioid doses, and provided more stable hemodynamics at the end of surgery. FIB showed higher efficiency and shortened hospital stay. Anesthesiologists should select appropriate block techniques based on the unique advantages of different nerve blocks and patients’ specific conditions.

## 1. Introduction

The hip joint plays a critical role in human motor function. Injuries or diseases affecting the hip joint can cause significant deterioration in motor skills and, in some cases, even lead to death. Hip arthroscopy has emerged as an important method for early detection and treatment of hip joint disorders. This technique has gained popularity in clinical practice due to its benefits, including smaller incisions and reduced damage to the joint cavity and surrounding soft tissue compared to open hip surgery.^[1]^ Total hip arthroplasty (THA) represents the conventional surgical remedy for hip fracture, a prevalent traumatic condition in the elderly. THA has evolved as the foremost technique for managing severe hip diseases, relieving hip pain, and reinstating hip function. Following THA, patients frequently suffer moderate to severe pain enduring several days. Such lingering pain commonly eventuates in deferred rehabilitation, and rigidity plus adhesion of hip-encompassing tissues.^[2]^ Suboptimal anesthetic management and postoperative care following hip surgery may substantially increase the incidence of adverse events and mortality.^[3,4]^ The perioperative management of patients, including anesthesia administration, is critical. Studies have demonstrated that the selection of appropriate anesthetic techniques can improve prognosis and reduce the incidence of adverse reactions.^[5,6]^

Regional anesthesia is an appealing option. However, the innervation of the hip joint is a complex process involving the femoral nerve (FN), obturator nerve (ON), and sciatic nerve. Additionally, the skin over the typical portal entry sites is innervated by the lateral femoral cutaneous nerve (LFCN).^[7]^ In light of the intricate innervation in this region, it is imperative to identify an optimal regional anesthesia technique that encompasses key attributes: ease of administration, rapid onset, potent analgesic efficacy, and minimal tissue disruption. Ultrasound-guided nerve block possesses the aforementioned advantages, and lumbar plexus block (LPB) and fascia iliaca block (FIB) have emerged as commonly employed techniques in hip joint surgery. LPB is a compartment block that targets FN, ON, and LFCN, making it a highly favorable option. Similarly, FIB is another compartment block technique utilized in clinical practice. Current research has shown that preoperative FIB combined with anesthesia is safe and effective, and can alleviate the pain of patients undergoing femoral shaft fracture surgery as well as emergency department hip fracture patients.^[8–10]^ The combination of LPB with general anesthesia has shown effectiveness in pain control, reducing postoperative opioid usage, and addressing related side effects in hip arthroplasty.^[11–13]^ It not only enhances intraoperative and postoperative analgesia effectively but also improves lower limb relaxation and immobility during hip arthroplasty, fracture repair, and other hip and knee joint surgeries.^[13,14]^

Despite the potential of both FIB and LPB in enhancing pain management, current studies suffer from limitations such as single-center design, small sample sizes, and inadequate outcome measures. Moreover, inconsistencies exist in the results of available studies, particularly regarding postoperative analgesic effects and intraoperative anesthetic doses. These limitations hinder anesthesiologists’ ability to determine the optimal approach. This meta-analysis aims to comprehensively evaluate the advantages and disadvantages of FIB and LPB before general anesthesia in hip surgery. Based on previous research evidence and clinical observations, we propose the following hypotheses: firstly, FIB reduces block procedure time and causes fewer adverse reactions; secondly, LPB demonstrates better anesthetic effect; thirdly, LPB reduces the use of general anesthetics and shortens hospital stay. This study will systematically retrieve and strictly screen included literature, using fixed-effect or random-effect models for data pooling to verify the above hypotheses, thereby assisting in the selection of nerve blocks for such patients.

## 2. Materials and methods

The study has been registered in PROSPERO with the ID number CRD42022365917. This study does not involve ethical approval or patients’ informed consent.

### 2.1. Strategy of literature search

PubMed, Web of Science, Cochrane Library, Embase, and China National Knowledge Infrastructure were searched for studies on FIB and LPB from database establishment until October 4, 2022. The search phrases (lumbar plexus OR LPB OR LPB) AND (FIB OR fascia iliaca compartment block OR FIB OR FICB) AND (hip OR THA) were created by a combination of subject terms, free words, and Boolean logical operators. To find possible studies that matched the inclusion criteria, a manual search of pertinent reviews and the references of the included literature was conducted.

### 2.2. Criteria of literature inclusion and exclusion

The following were the inclusion requirements: The type of literature was randomized controlled trial (RCT) study published nationally and internationally; treatment of the study subjects was surgery on the hip; the study subjects were randomly divided into 2 groups, and different groups received different methods of nerve block (FIB or LPB).

Studies were excluded if they met one of the following criteria: Abnormal or missing data; duplicate reports, conference reports, and reviews; the study’s subject is not human; low quality of literature (high risk of bias (ROB) item ≥3).

### 2.3. Extraction of data and quality assessment

The retrieved literature was independently screened by 2 authors according to the inclusion and exclusion criteria, and the following data information was extracted: author’s name, publishing year, literature’s source, fundamental traits of included cases, and important observation index (the difference outcome index caused by different nerve block methods). If the opinions of 2 reviewing authors differ, they debate. If there was still disagreement after discussion, a third-party opinion was sought.

The Cochrane ROB tool was used to assess the quality of the included literature. Cochrane ROB tool includes 7 aspects of bias risk assessment: Random sequence generation (selection bias); allocation concealment (selection bias); blinding of participants and personnel (performance bias); blinding of outcome assessment (detection bias); incomplete outcome data (attrition bias); selective reporting (reporting bias); other bias.^[15]^ Each bias risk is divided into 3 levels: “low risk,” “unclear”, and “high risk.” Literature with more than 3 high-risk bias items was regarded as low-quality literature.

### 2.4. Analysis of data

The average and standard deviation of the data expressed in median and range were estimated.^[16,17]^ Statistical analysis was performed using Reviewer Manager 5.4.1 software. The heterogeneity of the collected literature was assessed by *I*^2^, and the fixed-effects model was applied when *P* > .05 and *I*^2^ < 50%; otherwise, the random-effects model was applied. For dichotomous variables, relative risk (RR) and its 95% confidence interval (CI) were calculated. As for the measured data, the standardized mean difference (SMD) and its 95% CI were calculated, and differences were considered statistically significant when *P* ≤ .05. By computing the RR and 95% CI for both fixed-effects and random-effects models and contrasting the outcomes of the 2 groups, sensitivity analysis was carried out. The consolidated result was regarded as stable if there was no significant change following the model modification (no opposing conclusion was reached after altering the model).

### 2.5. Definition of observation index

Ultrasound imaging time: the time from the beginning to finding the puncture point.

Puncture time: the time from the needle entering the skin to reaching the injection point.

The time to take effect: Within 30 minutes after the injection of local anesthesia, sensory blockade is assessed every 5 minutes using cold stimulation and needle puncture methods. The sensory blockade is compared to the unaffected side using the following grading system: Grade 0 indicates sensitivity to cold stimulation and needle puncture; Grade 1 indicates reduced sensitivity to cold stimulation; Grade 2 indicates no sensation to cold stimulation but sensation to needle puncture; Grade 3 indicates no sensation to both cold stimulation and needle puncture. Effective blockade is defined as achieving Grade 2 in the regions innervated by the FN, LFCN, and ON, and the time to take effect is calculated.

Blood pressure and heart rate during operation: recordings are taken at 3 time points: laryngeal mask implantation, cutting skin, and suturing skin.

Visual analogue scale (VAS) is a widely used method for pain assessment in clinical practice. It involves using a movable ruler with a length of approximately 10 cm. One side of the ruler is marked with 10 scales, with “0” representing no pain and “10” representing the most intense and unbearable pain. In this study, VAS scores will be recorded at specific time points after surgery: 1, 6, 8, 12, 24, and 48 hours. The patients will be asked to indicate their level of pain by marking a point on the ruler corresponding to their perceived pain intensity. The distance from the “0” end of the ruler to the marked point will be measured and recorded as the VAS score. This allows for quantitative assessment of pain levels at different time points post-surgery.

Patient-controlled analgesia (PCA) is a method of pain management that allows patients to control their pain medication administration. Medications such as morphine or sufentanil are commonly used in PCA. The patient can press a button to self-administer the medication when they experience pain or discomfort.

The doses of sufentanil and remifentanil during operation: specifically refers to the doses used intravenously during operation.

Adverse events: nausea, vomiting, urinary retention, lethargy, itching, sensory or motor dysfunction, allergy and other side effects that may be caused by drugs during postoperative hospitalization.

## 3. Results

### 3.1. Literature search

**Figure 1.**
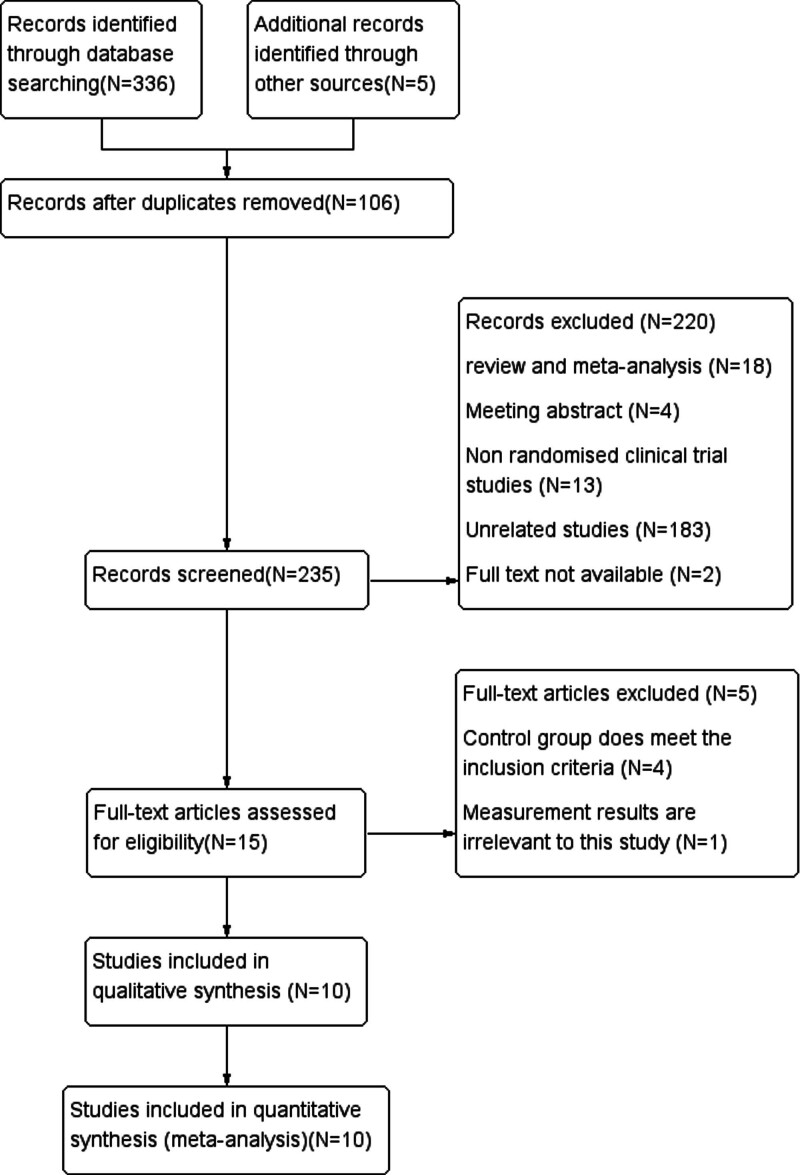
Study flow diagram.

The database was first screened for 336 publications using the search strategy, after which 15 papers were chosen based on features such as title, abstract, and keywords. Finally, 10 papers^[18–27]^ – 2 in English and 8 in Chinese – were further vetted by reading the full text (Fig. 1).

### 3.2. Baseline features

**Figure 2.**
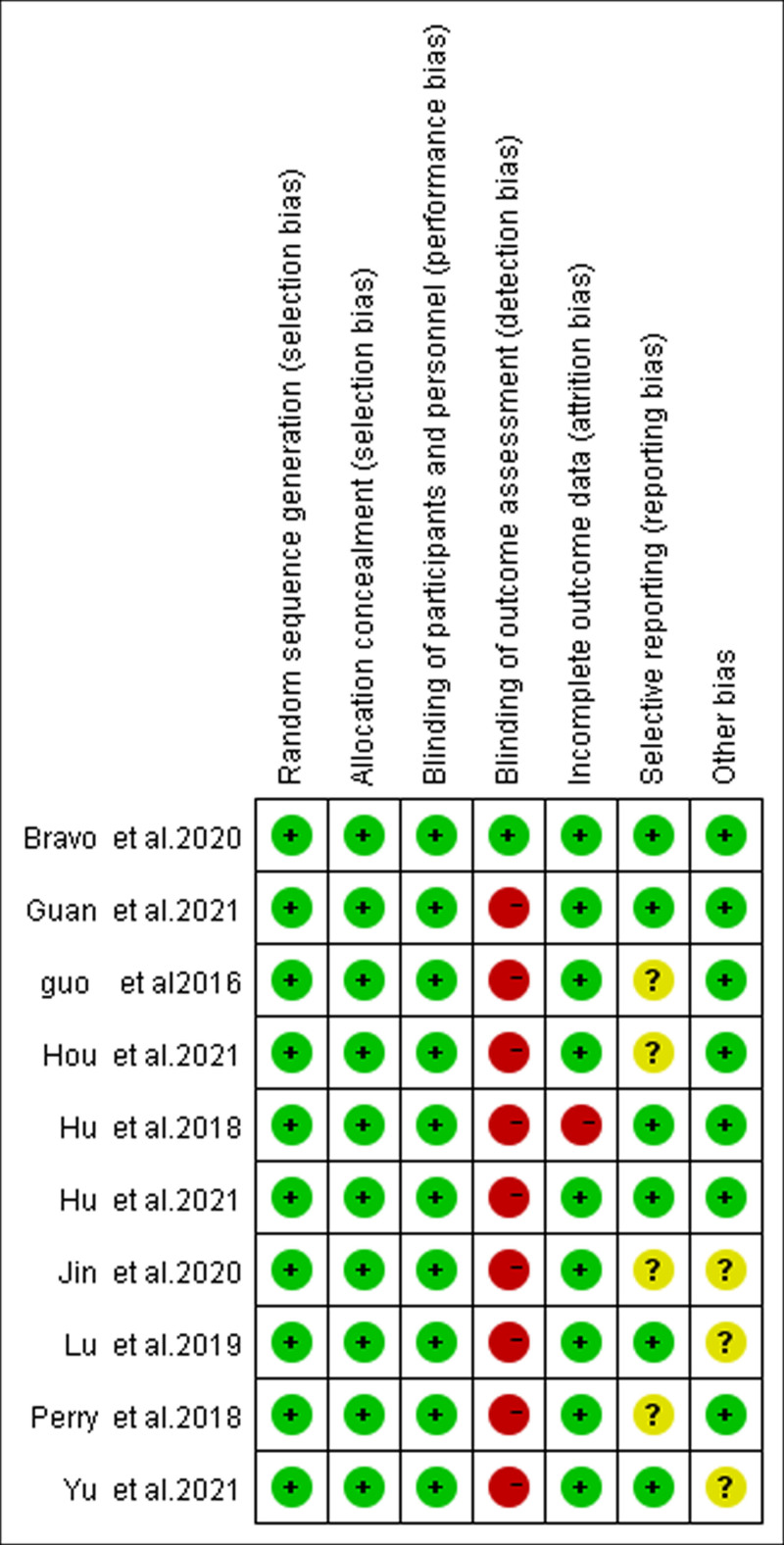
Risk of bias summary: review authors’ judgements about each risk of bias item for each included study.

Our analysis includes 10 research articles published between 2016 and 2021, all of which were RCTs. A total of 639 patients were enrolled, including 323 patients in FIB group (experimental group) and 316 patients in LPB group (control group), and 21 observational indices were extracted to enable comparison (Table 1). The quality of the 10 papers was evaluated by the Cochrane ROB tool, indicating that all papers show random methods, only one study was blinded to the investigator, and in one study 7 patients withdrew due to unsuccessful LPB (Fig. 2). All studies included LPB or FIB before general anesthesia, but were not grouped according to different approaches to FIB. All comparative results below use the LPB group as the reference group.

**Table 1:** Characteristics of the included studies.

Study	Year	Area	Sample size		Type and dose of local anesthetics	Outcome data from the study
			FIB group	LPB group		
Bravo et al^[18]^	2020	Chile	30	30	0.25% levobupivacaine 40 mL	08, 16, 17
Yu et al^[19]^	2021	China	30	30	0.4% ropivacaine 40 mL	01, 02, 07, 09, 12, 13, 15, 16
Lu et al^[20]^	2019	China	30	30	0.5% ropivacaine 30 mL	04, 05, 06, 09, 11, 13, 14, 16
Jin et al^[21]^	2020	China	30	30	0.4% ropivacaine 25 mL	02, 03, 04, 05, 16
Hu et al^[22]^	2021	China	33	33	0.25% ropivacaine 40 mL	01, 02, 10, 12, 14, 15, 16
Hu et al^[23]^	2018	China	50	43	0.375% ropivacaine 30 mL	06, 10, 12, 13, 14, 15, 16
Hou et al^[24]^	2021	China	35	35	0.25% ropivacaine 40 mL	09, 11, 13, 14, 15, 16
Guan et al^[25]^	2021	China	30	30	0.375% ropivacaine 40 mL	02, 03, 07, 09, 16
Guo et al^[26]^	2016	China	30	30	0.4% ropivacaine 35 mL	02, 08, 16, 17
Perry et al^[27]^	2018	New Zealand	25	25	0.3% ropivicaine 50 mL	16

01: Time of ultrasonic imaging, 02: Time of puncture, 03: Time to take effect, 04: Heart rate during operation, 05: Blood pressure during operation, 06: Doses of sufentanil during operation, 07: Doses of remifentanil during operation, 08: Time to first use of PCA, 09: Doses of sufentanil within 24 h after operation, 10–15: VAS score of postoperative 1, 6, 8, 12, 24, 48 h, 16: Incidence of adverse events, 17: Length of stay.

FIB = fascia iliaca block, LPB = lumbar plexus block, PCA = patient controlled analgesia, VAS = visual analogue scale (all data was VAS score at rest).

### 3.3. Comparison of the time of ultrasonic imaging

**Figure 3.**
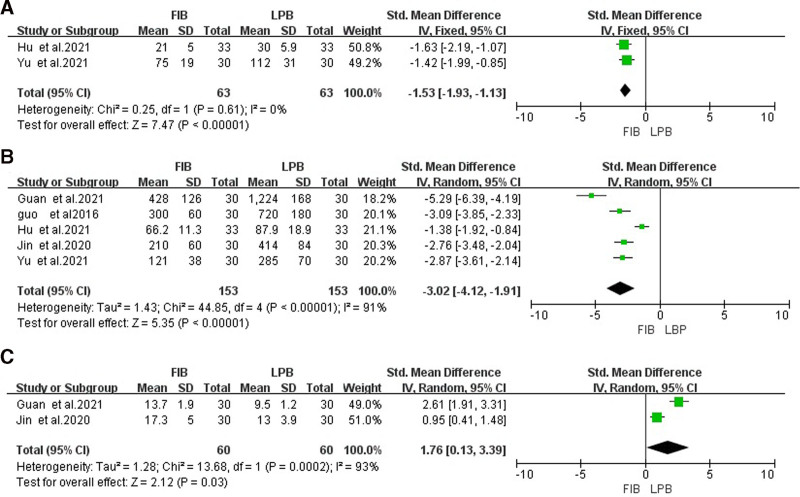
Forest map of comparison of the time of ultrasonic imaging (A), the time of puncture (B), the time to take effect (C). LPB group as the reference group. LPB = lumbar plexus block.

Two studies^[19,22]^ compared the time of ultrasonic imaging, and heterogeneity test showed that there was no heterogeneity (*P* > .05 and *I*^2^ < 50%), so the fixed-effects model analysis was selected. Meta-analysis results showed that the ultrasound imaging time in FIB was significantly lower than LPB [SMD = −1.53, 95% CI (−1.93 to −1.13), *P* < .001], as shown in Figure 3A.

### 3.4. Comparison of the time of puncture

Five studies^[19,21,22,25,26]^ compared the time of puncture, and heterogeneity test indicated there was heterogeneity (*P* < .05 and *I*^2^ > 50%), so the random-effects model analysis was selected. Meta-analysis results showed that the puncture time in FIB was significantly lower than LPB [SMD = −3.02, 95% CI (−4.12 to −1.91), *P* < .001], as shown in Figure 3B.

### 3.5. Comparison of the time to take effect

Two studies^[21,25]^ compared the time to take effect, and heterogeneity test indicated there was heterogeneity (*P* < .05 and *I*^2^ > 50%), so the random-effects model analysis was selected. Meta-analysis results showed that the time to take effect in FIB was significantly higher than LPB [SMD = 1.76, 95% CI (0.13–3.39), *P* = .03], as shown in Figure 3C.

### 3.6. Comparison of heart rate during operation

Two studies^[20,21]^ compared the heart rate during operation, the same recording time includes laryngeal mask implantation, cutting skin, and suturing skin. The details are as follows:

#### 3.6.1. Heart rate during laryngeal mask implantation

**Figure 4.**
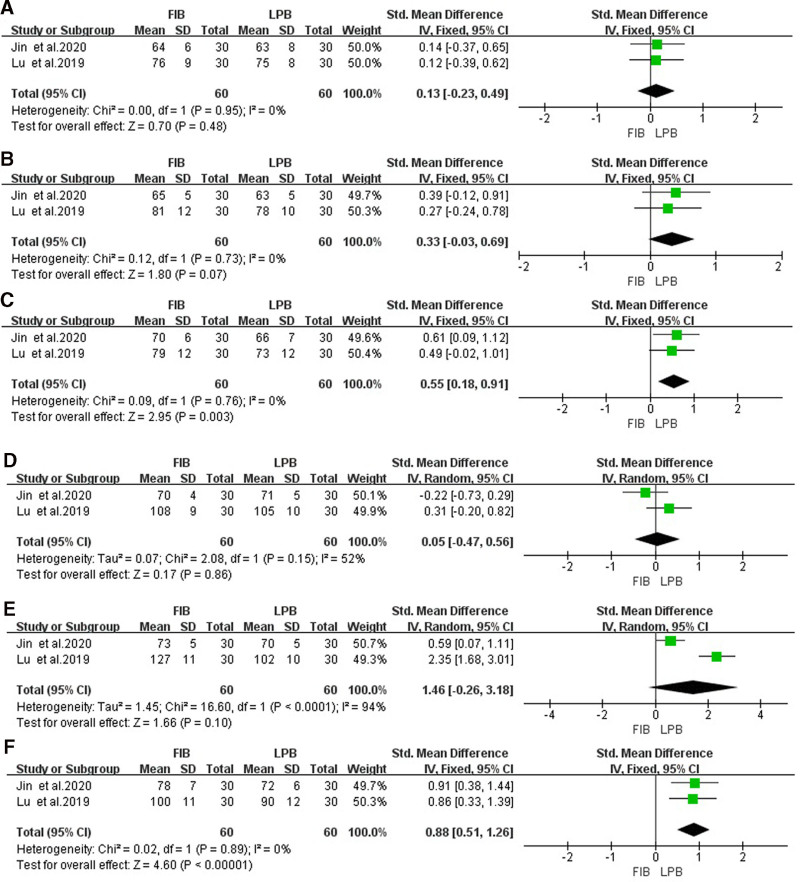
Forest map of comparison of heart rate during laryngeal mask implantation (A), heart rate during cutting skin (B), heart rate during suturing skin (C), blood pressure during laryngeal mask implantation (D), blood pressure during cutting skin (E), blood pressure during suturing skin (F). LPB group as the reference group. LPB = lumbar plexus block.

The heterogeneity test showed that there was no heterogeneity (*P* > .05 and *I*^2^ < 50%), so the fixed-effects model analysis was selected. Meta-analysis results showed that there was no significant difference in heart rate during laryngeal mask implantation between the 2 groups [SMD = 0.13, 95% CI (−0.23 to 0.49), *P* = .48], as shown in Figure 4A.

#### 3.6.2. Heart rate during cutting skin

The heterogeneity test showed that there was no heterogeneity (*P* > .05 and *I*^2^ < 50%), so the fixed-effects model analysis was selected. Meta-analysis results showed that there was no significant difference in heart rate during cutting skin between the 2 groups [SMD = 0.33, 95% CI (−0.03 to 0.69), *P* = .07], as shown in Figure 4B.

#### 3.6.3. Heart rate during suturing skin

The heterogeneity test showed that there was no heterogeneity (*P* > .05 and *I*^2^ < 50%), so the fixed-effects model analysis was selected. Meta-analysis results showed that heart rate during suturing skin in the FIB group was significantly higher than that in the LPB group [SMD = 0.55, 95% CI (0.18–0.91), *P* = .03], as shown in Figure 4C.

### 3.7. Comparison of blood pressure during operation

Two studies^[20,21]^ compared the blood pressure during operation, the same recording time includes during laryngeal mask implantation, cutting skin, and suturing skin. The details are as follows:

#### 3.7.1. Blood pressure during laryngeal mask implantation

The heterogeneity test indicated there was heterogeneity (*I*^2^ > 50%), so the random-effects model analysis was selected. Meta-analysis results showed no significant difference in blood pressure during laryngeal mask implantation between the 2 groups [SMD = 0.05, 95% CI (−0.47 to 0.56), *P* = .80], as shown in Figure 4D.

#### 3.7.2. Blood pressure during cutting the skin

The heterogeneity test indicated there was heterogeneity (*P* < .05 and *I*^2^ > 50%), so the random-effects model analysis was selected. Meta-analysis results showed no significant difference in blood pressure during cutting skin between the 2 groups [SMD = 1.46, 95% CI (−0.26 to 3.18), *P* = .10], as shown in Figure 4E.

#### 3.7.3. Blood pressure during suturing skin

The heterogeneity test showed that there was no heterogeneity (*P* > .05 and *I*^2^ < 50%), so the fixed-effects model analysis was selected. Meta-analysis results showed that blood pressure during suturing skin in the FIB group was significantly higher than LPB [SMD = 0.88, 95% CI (0.51–1.26), *P* < .001], as shown in Figure 4F.

### 3.8. Comparison of VAS score

#### 3.8.1. VAS score of postoperative 1 hour

**Figure 5.**
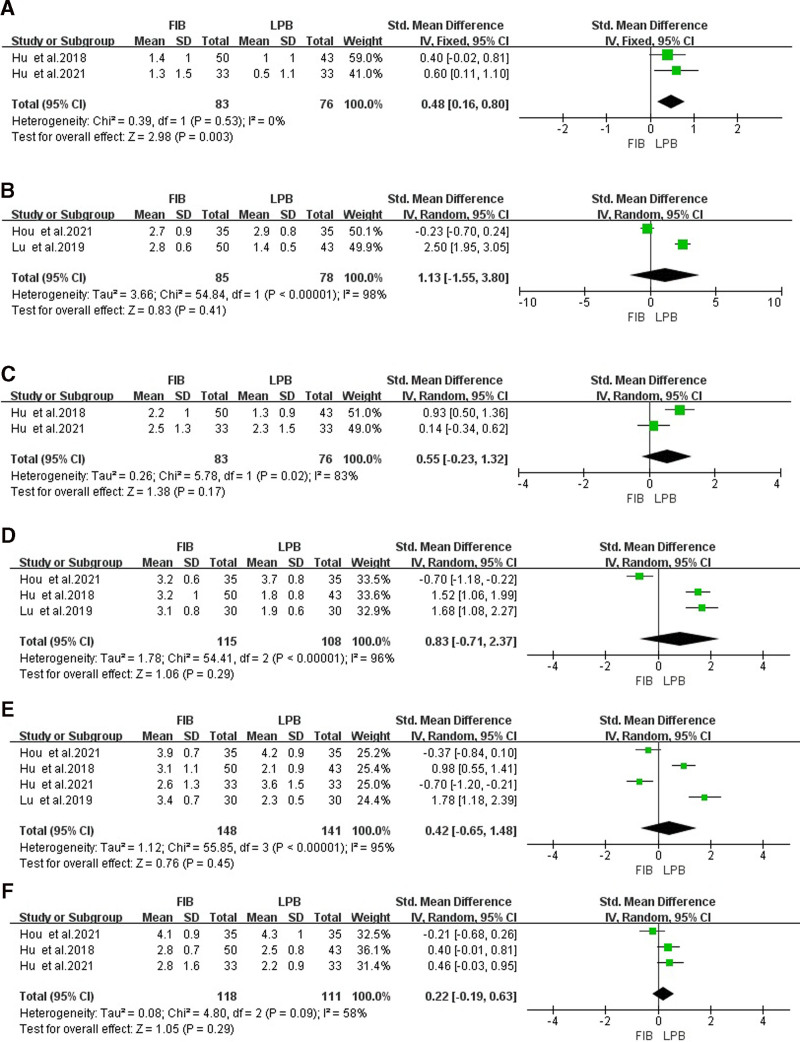
Forest map of comparison of VAS score of postoperative 1, 6, 8,12, 24, 48 h (A–F). LPB group as the reference group. LPB = lumbar plexus block, VAS = visual analogue scale.

Two studies^[22,23]^ compared the VAS score of postoperative 1 hour, and the heterogeneity test showed no heterogeneity (*P* > .05 and *I*^2^ < 50%), so the fixed-effects model analysis was selected. Meta-analysis results showed that the VAS score of postoperative 1 hour in FIB was significantly higher than LPB [SMD = 0.48, 95% CI (0.16–0.80), *P* = .003], as shown in Figure 5A.

#### 3.8.2. VAS score of postoperative 6 hours

Two studies^[20,24]^ compared the VAS score of postoperative 6 hours, and the heterogeneity test indicated there was heterogeneity (*P* < .05 and *I*^2^ > 50%), so the random-effects model analysis was selected. Meta-analysis results showed no significant difference in the VAS score of postoperative 6 hours between 2 groups [SMD = 1.13, 95% CI (−1.55 to −3.80), *P* = .41], as shown in Figure 5B.

#### 3.8.3. VAS score of postoperative 8 hours

Two studies^[22,23]^ compared the VAS score of postoperative 8 hours, and the heterogeneity test indicated there was heterogeneity (*P* < .05 and *I*^2^ > 50%), so the random-effects model analysis was selected. Meta-analysis results showed no significant difference in the VAS score of postoperative 8 hours between 2 groups [SMD = 0.55, 95% CI (−0.23 to 1.32), *P* = .17], as shown in Figure 5C.

#### 3.8.4. VAS score of postoperative 12 hours

Three studies^[20,23,24]^ compared the VAS score of postoperative 12 hours, and the heterogeneity test indicated there was heterogeneity (*P* < .05 and *I*^2^ > 50%), so the random-effects model analysis was selected. Meta-analysis results showed no significant difference in VAS score of postoperative 12 hours between 2 groups [SMD = 0.83, 95% CI (−0.71 to 2.37), *P* = .29], as shown in Figure 5D.

#### 3.8.5. VAS score of postoperative 24 hours

Four studies^[20,22–24]^ compared the VAS score of postoperative 24 hours, and the heterogeneity test indicated there was heterogeneity (*P* < .05 and *I*^2^ > 50%), so the random-effects model analysis was selected. Meta-analysis results showed no significant difference in VAS score of postoperative 24 hours between 2 groups [SMD = 0.42, 95% CI (−0.65 to 1.48), *P* = .45], as shown in Figure 5E.

#### 3.8.6. VAS score of postoperative 48 hours

Three studies^[22–24]^ compared the VAS score of postoperative 48 hours, and the heterogeneity test indicated there was heterogeneity (*I*^2^ > 50%), so the random-effects model analysis was selected. Meta-analysis results showed no significant difference in VAS score of postoperative 48 hours between 2 groups [SMD = 0.22, 95% CI (−0.19 to 0.63), *P* = .29], as shown in Figure 5F.

### 3.9. Comparison of the doses of sufentanil during operation

**Figure 6.**
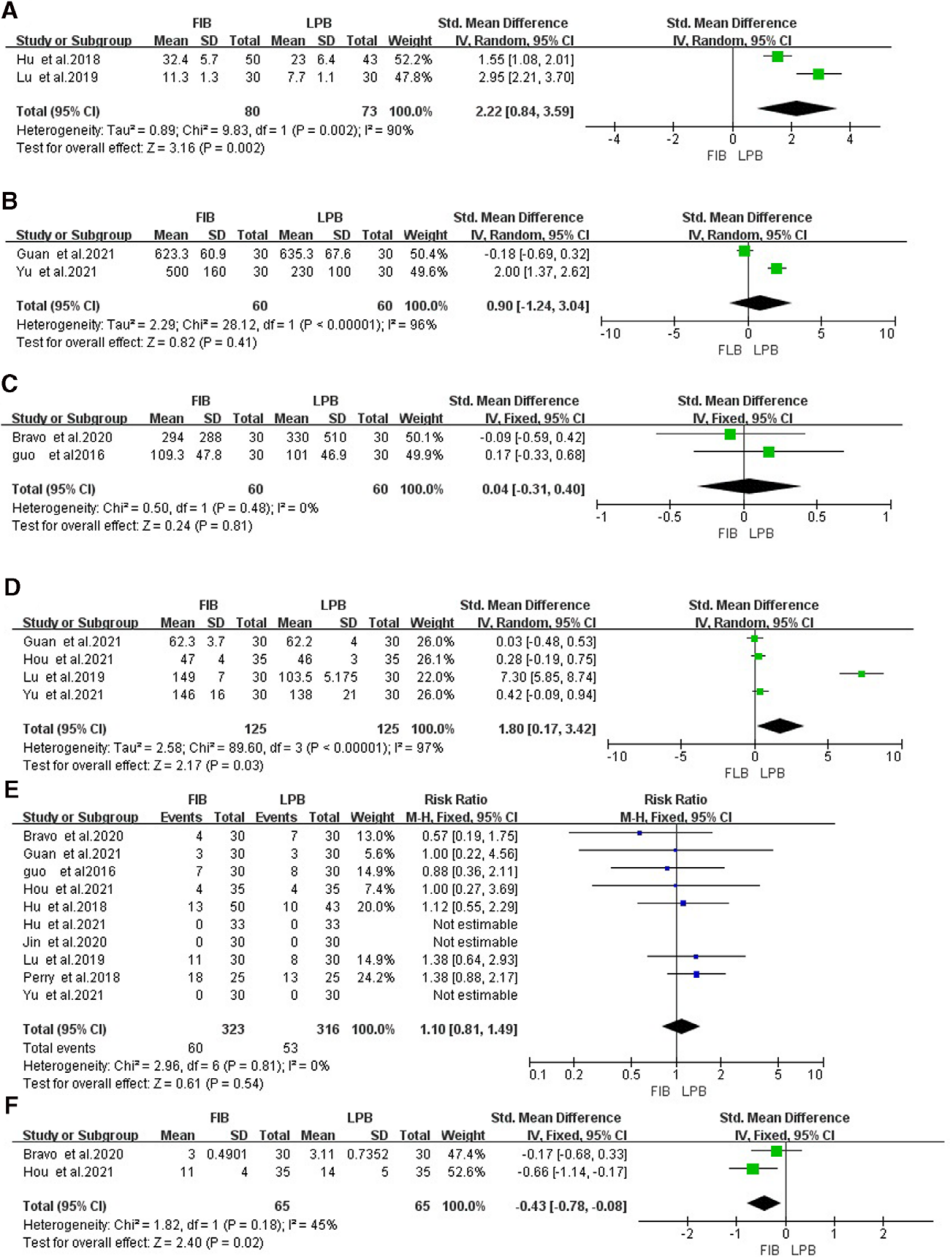
Forest map of comparison of the dosage of remifentanil during operation (A), the dosage of remifentanil during operation (B), the time to first use of PCA (C), the dosage of sufentanil within 24 h after operation (D), the incidence of adverse events (E), the length of stay (F). LPB group as the reference group. LPB = lumbar plexus block, PCA = patient-controlled analgesia.

Two studies^[20,23]^ compared the doses of sufentanil during operation, and the heterogeneity test indicated there was heterogeneity (*P* < .05 and *I*^2^ > 50%), so the random-effects model analysis was selected. Meta-analysis results showed that the doses of sufentanil during operation in FIB group were significantly higher than that in LPB [SMD = 2.22, 95% CI (0.84–3.59), *P* = .002], as shown in Figure 6A.

### 3.10. Comparison of the doses of remifentanil during operation

Two studies^[19,25]^ compared the doses of remifentanil during operation, and the heterogeneity test indicated there was heterogeneity (*P* < .05 and *I*^2^ > 50%), so the random-effects model analysis was selected. Meta-analysis results showed no significant difference in the use of remifentanil during operation between 2 groups [SMD = 0.90, 95% CI (−1.24 to 3.04), *P* = .41], as shown in Figure 6B.

### 3.11. Comparison of the time to first use of PCA

Two studies^[18,26]^ compared the time to first use of PCA, and the heterogeneity test showed that there was no heterogeneity (*P* > .05 and *I*^2^ < 50%), so the fixed-effects model analysis was selected. Meta-analysis results showed no significant difference in the time to first use of PCA between 2 groups [SMD = 0.04, 95% CI (−0.31 to 0.40), *P* = .81], as shown in Figure 6C.

### 3.12. Comparison of the doses of sufentanil within 24 hours after operation

Four studies^[19,20,24,25]^ compared the use of sufentanil within 24 hours after operation, and the heterogeneity test indicated heterogeneity (*P* < .05 and *I*^2^ > 50%), so the random-effects model analysis was selected. Meta-analysis results showed that the doses of sufentanil within 24 hours after operation in the FIB group were significantly higher than that in LPB [SMD = 1.80, 95% CI (0.17–3.42), *P* = .03], as shown in Figure 6D.

### 3.13. Comparison of the incidence of adverse events

All the studies compared the incidence of adverse events, and the heterogeneity test showed that there was no heterogeneity (*P* > .05 and *I*^2^ < 50%), so the fixed-effects model analysis was selected. Meta-analysis results showed no significant difference in the incidence of adverse events between the 2 groups [RR = 1.10, 95% CI (0.81–1.49), *P* = .81], as shown in Figure 6E.

### 3.14. Comparison of the length of stay

Two studies^[18,26]^ compared the length of stay, and the heterogeneity test showed that there was no heterogeneity (*P* > .05 and *I*^2^ < 50%), so the fixed-effects model analysis was selected. Meta-analysis results showed that the length of stay in the FIB group was significantly shorter than that in LPB [SMD = −0.43, 95% CI (−0.78 to −0.08), *P* = .02], as shown in Figure 6F.

## 4. Sensitivity analysis

Sensitivity analysis indicated that the meta-analysis findings were stable for all except 7 indexes: blood pressure during cutting skin, VAS scores of postoperative 6, 8, 12, 24 hours, the doses of remifentanil during operation, and the length of stay (Table 2).

**Table 2:** Meta-analysis results of important observation indexes for comparison between LPB and FIB in hip surgery.

Observation indexes	Included studies	Heterogeneity (χ^2^)	*P*	Fixed-effect model (FEM)	*P*	Random-effect model (REM)	*P*
Time consumed							
Ultrasonic imaging	2	0.25	.61	−1.53 [−1.93, −1.13]	<.001	−1.53 [−1.93, −1.13]	<.001
Puncture	5	44.85	<.001	−2.57 [−2.89, −2.25]	<.001	−3.02 [−4.12, −1.91]	<.001
Take effect	2	13.68	<.001	1.56 [1.14–1.99]	<.001	1.76 [0.13–3.39]	.03
Heart rate							
During laryngeal mask implantation	2	0	.95	0.13 [−0.23, 0.49]	.48	0.13 [−0.23, 0.49]	.48
During cutting skin	2	0.12	.73	0.33 [−0.03, 0.69]	.07	0.33 [−0.03, 0.69]	.07
During suturing skin	2	0.09	.76	0.55 [0.18–0.91]	.03	0.55 [0.18–0.91]	.03
Blood pressure							
During laryngeal mask implantation	2	2.08	.15	0.05 [−0.31, 0.41]	.80	−0.26 [−2.33, 1.81]	.80
During cutting skin	2	16.60	<.001	1.25 [0.84–1.66]	<.001	1.46 [−0.26, 3.18]	.10
During suturing skin	2	0.02	.89	0.88 [0.51–1.26]	<.001	0.88 [0.51–1.26]	<.001
VAS score							
Postoperative 1 h	2	0.39	.53	0.48 [0.16–0.80]	.003	0.48 [0.16–0.80]	.003
Postoperative 6 h	2	54.84	<.001	0.92 [0.57–1.28]	<.001	1.13 [−1.55, 3.80]	.41
Postoperative 8 h	2	5.78	.02	0.58 [0.26–0.90]	<.001	0.55 [−0.23, 1.32]	.17
Postoperative 12 h	3	54.41	<.001	0.75 [0.46–1.04]	<.001	0.83 [−0.71, 2.37]	.29
Postoperative 24 h	4	55.85	<.001	0.34 [0.09–0.59]	.007	0.42 [−0.65, 1.48]	.45
Postoperative 48 h	3	4.8	.09	0.23 [−0.03, 0.49]	.09	0.22 [−0.19, 0.63]	.29
Others							
Doses of sufentanil during operation	2	9.83	<.001	1.94 [1.55, 2.34]	<.001	2.22 [0.84, 3.59]	.002
Doses of remifentanil during operation	2	28.12	<.001	0.68 [0.28–1.07]	<.001	0.90 [−1.24, 3.04]	.41
Time to first use of PCA	2	0.50	.48	0.04 [−0.31, 0.40]	.81	0.04 [−0.31, 0.40]	.81
Doses of sufentanil within 24 h after operation	4	89.6	<.001	0.51 [0.23–0.79]	<.001	1.80 [0.17–3.42]	.03
Incidence of adverse events	10	2.96	.81	1.10 [0.81–1.49]	.81	1.16 [0.87–1.56]	.31
Length of stay	2	1.82	.18	−0.43 [−0.78, −0.08]	.02	−0.42 [−0.89, 0.05]	.08

With the LPB group as the reference group.

FIB = fascia iliaca block, LPB = lumbar plexus block, PCA = patient-controlled analgesia.

## 5. Discussion

Effective postoperative pain management is a critical component of modern joint surgery. Adult reconstructive surgeons widely recognize that a multimodal pain management approach, incorporating at least one form of regional anesthesia, is an indispensable part of reducing postoperative pain and maximizing patient satisfaction.^[28]^ In hip joint surgeries, there is still no consensus on the optimal regional anesthesia technique. FIB and LPB are both viable options for regional nerve blockade. As shown in Table 3, we compared FIB and LPB in various aspects from previous studies.

**Table 3:** Comparison between FIB and LPB.

	FIB	LPB
Main target nerves	FN, LFCN^[29]^	FN, LFCN, ON^[30]^
Unique complications	Inguinal and back pain, bruising, and inguinal hematoma^[29]^	Retroperitoneal or lumbar muscle hematoma^[31]^
Quadriceps weakness	Common, no difference in incidence between FIB and LPB^[18]^	Common, no difference in incidence between FIB and LPB^[18]^
Nerve injury	Rare^[32]^	Rare, as low as 0.1%^[33]^
Coverage	Thigh, knee, and hip regions^[34]^	Hip joint, anterior and medial compartments of the thigh, and femur^[30]^
Disadvantage	Risks of systemic poisoning due to intravascular injection^[35]^	Risks of intrathecal injection and spinal anesthesia^[31]^

FIB = fascia iliaca block, FN = femoral nerve, LFCN = lateral femoral cutaneous nerve, LPB = lumbar plexus block, ON = obturator nerve.

This study aimed to compare the advantages and disadvantages of these 2 regional anesthesia techniques in hip surgery. The results indicated that FIB had advantages in terms of ultrasound imaging time, puncture time, and length of stay. On the other hand, LPB demonstrated advantages in terms of time to take effect, heart rate and blood pressure at the end of the operation, doses of sufentanil during and within 24 hours after the operation, time to first use of PCA, as well as VAS score at postoperative 1 hour. The laryngeal mask airway plays a pivotal role in the procedure, and the process of inserting the laryngeal mask induces noticeable hemodynamic changes when general anesthesia is insufficient. However, we did not observe significant hemodynamic variations during the placement of the laryngeal mask, indicating consistent depth of general anesthesia across both groups. Consequently, the improved hemodynamics in the LPB group suggest a more effective peripheral nerve block during skin suturing. Additionally, the lower VAS score at 1 hour postoperatively and reduced use of analgesic medication further underscore the advantages of LPB.

From the perspective of anatomical structure, the lumbar plexus is located deep within the paravertebral muscles, making it more challenging to visualize with ultrasound imaging. Additionally, the surrounding tissue in this area is rich in nerves and blood vessels, requiring more time for the operator. On the other hand, the fascia iliaca compartment is more superficial, allowing for clearer ultrasound imaging of the surrounding blood vessels. Consequently, it can be imaged and injected more quickly. The hip is innervated predominately by branches of the lumbar plexus: the FN, ON, and the LFCN; as well as the sacral plexus via the nerve to the quadratus femoris, and at times, directly via the sciatic nerve.^[36]^ We observed that at the end of the operation, postoperative 1 hours and the first requirement of PCA, LPB had more advantages, which may be attributed to the more complete and proximal nerve blockade. In addition to the blockade of the FN and lateral cutaneous nerve, LPB also provides anesthesia to other nerves originating from the lumbar plexus. The most important of these is the ON, which transmits sensory information from the hip joint, adductor muscles, medial aspect of the femur, and inner thigh skin.^[13]^ It is not uncommon for the accessory ON, originating from the lumbar plexus, to innervate the anterior capsule of the hip joint.^[36]^ These nerves are not anesthetized by FIB. Postoperative pain after hip surgery reaches its peak at 1 to 2 hours postoperatively,^[37]^ and the analgesic advantage of LPB is mainly evident during the peak pain period, specifically at postoperative 1 hour, with no statistically significant differences observed at postoperative 6, 8, 12, 24, and 48 hours.

In our study, there was no significant difference in the overall incidence of adverse events between the 2 groups. This is because the main adverse reactions reported in the included literature were postoperative nausea and vomiting, and there were no reports of serious adverse events. However, we should also be aware of some potential risks, such as falls due to transient quadriceps muscle paralysis following nerve blockade,^[38]^ which can lead to catastrophic consequences such as fractures and reoperations. Additionally, there are potential risks associated with intravascular administration, such as seizures or arrhythmias. Furthermore, LPB has unique severe complications, such as complete spinal cord block,^[39]^ although it is rare and should not be completely disregarded.

Clinical practitioners need to consider the influence of surgical techniques on the choice of anesthesia methods. For instance, in the commonly performed proximal femoral nail antirotation procedure, which typically does not involve the joint capsule, FIB can provide effective anesthesia. The selection between the suprainguinal and infrainguinal approaches for FIB should not be ignored. Generally, the suprainguinal approach provides anesthesia to the anterior thigh, knee, and distribution of the saphenous nerve, while the infrainguinal approach covers the anterior thigh and lateral aspect of the thigh. Previous research has demonstrated that the suprainguinal approach achieves better anesthesia efficacy in hip surgeries,^[40]^ because cephalic spread of the local anesthetic is more.^[41]^ LPB requires the patient to be in a lateral position, while FIB can be performed in a supine position. However, considering that some patients undergoing hip surgery may have difficulty being in a lateral position due to fractures, the choice between LPB and FIB should also be carefully considered.

The literature we included featured various nerve blocking drugs, such as levobupivacaine and ropivacaine, with studies utilizing different concentrations and doses. Research^[42]^ has indicated that levobupivacaine generally exhibits a 50% higher potential in suppressing tetrodotoxin-resistant sodium ion channels compared to ropivacaine. However, studies have also demonstrated that there is no discernible difference in analgesic quality at equivalent doses, namely levobupivacaine 0.25% and ropivacaine 0.375%.^[43]^ Despite varying conclusions across studies, we utilized meta-analysis to weigh the final results of each study regarding nerve block drugs. Our focus remained on comparing the disparities between the FIB and LPB techniques, which, to some extent, mitigated the influence of different drugs. While a stratified analysis based on different drugs, doses, and concentrations would enhance the reliability of the results, we acknowledge limitations in the available literature prevented us from completing this task. Sensitivity analysis reveals the instability of multiple results, which can be attributed to several factors. Firstly, the lack of relevant literature contributes to this instability. Secondly, in Bravo Study,^[18]^ the mean and standard deviation were estimated using the median and range, introducing some variability. Finally, among the unstable results, the majority can be attributed to the subjective nature of the VAS ratings, making their instability somewhat inevitable. To enhance the stability and credibility of the results, future research endeavors should encompass a broader range of literature sources for data fitting and stratified analysis.

## 6. Limitations

There are various limitations to this study: Due to the scarcity of domestic and foreign studies comparing FIB and LPB, as well as the variable quality of the literature, 10 papers were screened precisely according to the inclusion and exclusion criteria, although some publishing bias may still exist; limited by the small number of included studies, certain biases (such as unadjusted bias and publication bias) were not properly addressed. When sufficiently more similar studies are included in the future, these biases can be detected and corrected through methods like meta-regression analysis and Begg test; because of the scarcity of literature, the observation indicators in this study were not adequately merged, which may have an impact on the development of the final results; existing researches are insufficiently in-depth, and more important prospective cohort studies are lacking. As a result, more rigorous designs, large sample size, and multicenter studies are required to evaluate the benefits and drawbacks of FIB and LPB.

## 7. Conclusion

It is evident that LPB has a shorter onset time and provides better analgesic effects, leading to a significant reduction in opioid usage during and after surgery. However, LPB does present challenges such as longer ultrasound imaging and puncture times, which may be difficult for operators. Additionally, it can result in prolonged bed rest and delayed rehabilitation exercises, leading to extended hospital stays. It is important to note that LPB is not suitable for patients undergoing surgery in a lateral position.

We suggest that clinical practitioners should assist patients in making personalized choices based on their specific circumstances. For patients who require a shorter onset time, have higher analgesic needs, and wish to minimize opioid usage, LPB may be a more suitable option. On the other hand, FIB may be preferable for patients who require shorter hospital stays, are at a higher risk of hospital-acquired infections, or are being treated in facilities without accurate ultrasound equipment. It is crucial to consider the surgical approach and the anesthesia doctor’s proficiency in both techniques and to integrate them with other anesthesia methods to select the most favorable plan for each patient comprehensively. Moreover, further research is needed to exhaustively compare and analyze the advantages and disadvantages of each technique in terms of comfort and recovery for hip surgery patients.

## Author contributions

**Data curation:** Hongxia Mou.

**Investigation:** Jing Wu, Hongxia Mou, Xiaowei Luo.

**Methodology:** Jing Wu, Hongxia Mou, Xiaowei Luo.

**Project administration:** Hongxia Mou.

**Software:** Hongxia Mou.

**Supervision:** Xiaowei Luo.

**Validation:** Jing Wu.

**Writing – original draft:** Jing Wu.

**Writing – review & editing:** Xiaowei Luo.
